# Nutrient Composition of Retail Samples of Australian Beef Sausages

**DOI:** 10.3390/nu7115491

**Published:** 2015-11-19

**Authors:** Judy Cunningham, Van Nguyen, Paul Adorno, Veronique Droulez

**Affiliations:** 1 42 Caroline Street, Annerley 4103 QLD, Australia; judyc121@gmail.com; 2 Meat & Livestock Australia, Locked Bag 991, North Sydney 2059 NSW, Australia; vnguyen@mla.com.au; 3 National Measurement Institute, 153 Bertie Street, Port Melbourne 3207 VIC, Australia; paul.adorno@measurement.gov.au

**Keywords:** nutrient, beef, sausage, composition, retail, Australia, red meat

## Abstract

Some nutrient data for beef sausages in Australia’s food composition table, NUTTAB 2010, is over 25 years old and may no longer reflect the composition of this popular food. To update this, 41 retail samples of fresh beef sausages were purchased in Melbourne, Australia, in May 2015. Each purchase was analysed, uncooked, for moisture, protein and fat. Sausages were then grouped by fat content into one of three composites and analysed for a wide range of nutrients, before and after dry heat cooking, the most popular sausage cooking method. Fat content in raw sausages averaged 14.9 g/100 g, 30% lower than NUTTAB values, varying from 7.3 to 22.6 g/100 g. This indicates it is possible to formulate leaner sausages that meet consumer expectations and may qualify for certain nutrition labelling statements. Under current Australian labelling requirements, two low fat sausages contain sufficient protein, B12, niacin, phosphorus and zinc to qualify as a good source of these nutrients and sufficient iron, selenium and vitamin A to qualify as a source of these. Sodium levels are higher than fresh beef, ranging from 680 to 840 mg/100 g. These data will be used to update NUTTAB and support product labelling and consumer education.

## 1. Introduction

Sausages are a popular food in Australia featuring about once a fortnight in the main meal repertoire [1]. In the most recent national nutrition survey of Australians, in 2011–2013, 6.3% of those surveyed reported having eaten fresh, regular fat sausages on the day of the survey, with smaller proportions eating reduced fat sausages (0.5%) and mixed dishes containing sausages (0.2%). For regular fat sausages, mean per capita consumption was estimated at 9.1 g/day. When Australians ate sausages, median consumption was 101 g/day [2].

Sausage composition is regulated under the Australia New Zealand Food Standards Code [3], where they are defined as meat that is minced or comminuted, which may be combined with other foods, and then encased or formed into discrete units. They must contain no less than 500 g/kg fat free meat flesh and the fat content must be no more than half of the amount of the fat free meat flesh. Fresh sausages sold in Australia are not fermented, cured or dried, are sold raw and typically contain sulphites as preservatives. They do not contain added nitrites or nitrates as this is not permitted in sausages containing raw, unprocessed meat in Australia [3,4]. A range of meats are used to make sausages, with beef, lamb, pork, chicken and kangaroo sausages typically available for sale. This study focussed on beef sausages, which appear to be the most common [5].

Up-to-date nutrient composition data representative of foods available for purchase and prepared using typical preparation practices in Australia is required to support food labelling, nutrition research and dietetic practice. Food Standards Australia New Zealand’s food and nutrient database, NUTTAB (Nutritional Data Tables for Australia) 2010 [6], contains nutrient data for 2668 foods available in Australia, including beef sausages (raw, grilled and fried). The majority of nutrient data for raw and pan fried beef sausages were generated on samples collected in 1988, while data for grilled beef sausages is from samples collected in 2006 [7]. Since 1988, a wider variety of meat products have become available for purchase, including a trend towards leaner options [8]. It is important that food and nutrient databases reflect the variety of product choices available for consumption as well as contemporary cooking practices.

The aims of this study were: (1) to understand the variation in moisture, fat and protein content of different types of beef sausages available for purchase in Australian retail outlets; (2) to investigate the effect of the most common cooking methods on levels of these nutrients; and (3) to determine the content of a wide range of proximate nutrients, vitamins, minerals, fatty acids and cholesterol in representative composite samples of raw and cooked beef sausages. This new data will be used to update existing data held in NUTTAB 2010 and to support product labelling and consumer education.

## 2. Experimental Section

### 2.1. Sampling Location

Beef sausages available for purchase from representative retail outlets in Melbourne, Australia, were purchased in May 2015. Melbourne is Australia’s second largest city and was selected as the sampling location due to its proximity to the laboratory conducting analyses. Previous surveys of the nutrient composition of Australian foods have not shown consistent effects of purchase location on nutrient composition [7] and a recent survey of Australian beef mince [9] did not find consistent effects of sampling location on nutrient content. Sausage production in national supermarket chains in Australia is largely centralised, with a limited number of production sites and widespread distribution from these sites. Sausages supplied by independent butchers are generally prepared onsite, often with sausage pre-mixes produced in central locations [10]. Samples were selected at nine stores reflecting the main places where Australians purchase sausages—major supermarket chains (3), independent supermarkets (3) and independent butchers (3). Shops were located in suburbs of low, middle and high socio-economic status [11].

### 2.2. Sampling Methods

Samplers were instructed to purchase samples of every type of beef sausage available at the outlet visited and to confirm the meat content of the sausages, from the label or by asking butchers. A total of 41 different types of beef sausages were collected; sausages containing meats other than beef were not selected. Each purchase was approximately 3 kg. Product descriptors were recorded, as was information on other ingredients used (where possible). Sausages were transported to the laboratory, chilled and in their original packaging, on the day of purchase or the next day.

### 2.3. Sample Preparation—Individual Raw Samples

At the laboratory, the mass of sausages from each purchase was determined. Approximately 250 g of each purchase was immediately homogenised in a heavy-duty blender, mixed with an equal mass of hot water to facilitate homogenisation, and transferred into plastic containers with screw top lids. The containers were filled to a minimum headspace and stored at 4 °C prior to analysis. Each container was labelled with a sample description and a unique Laboratory Registration Number. These samples were used for analysis of moisture, protein and fat content of individual raw samples.

The remaining sausages from each purchase were wrapped tightly in plastic film and frozen at −18 °C for further investigations.

### 2.4. Investigation of the Effect of Different Cooking Methods on Nutrient Composition

Additional amounts of one type of sausage were purchased for a preliminary study of the effect of common cooking methods on moisture, fat and protein in beef sausages. The sausage type selected for this was the biggest selling beef sausage in one major national supermarket chain. Three cooking methods believed to be the most commonly used were selected. These were: pan-frying for 15 min in a Teflon-coated steel pan, with a light spray of cooking oil, over a medium heat setting; barbecuing on a cast-iron flat plate for 15 min on a medium heat setting without cooking oil; or simmering in water for 10 min. For each cooking method, 11 sausages were cooked by a qualified home economist, who was asked to cook the samples as they would commonly be cooked in the home. The internal temperature of one sausage from each cooking method was recorded and ranged from 83 °C to 90 °C. Samples were weighed before and after cooking. After cooling, cooked sausages were packaged in airtight plastic bags with minimal headspace, refrigerated and subsequently homogenized in a heavy duty blender for analysis of moisture, protein and fat content.

### 2.5. Sample Preparation—Composite Samples

After consideration of the moisture, protein and fat contents in individual samples, sausages were grouped into one of three groups based on total fat content in raw sausages—lower fat (mean fat content approximately 10 g/100 g); medium fat (mean fat content approximately 14 g/100 g); and higher fat (mean fat content approximately 19 g/100 g). Composite samples containing equal masses of each sausage type were then prepared based on this grouping. Where sausages needed to be cut to achieve equal aliquots, a portion was cut from the middle of a sausage.

The cooking methods investigated in this phase were selected after an online omnibus survey of a nationally representative sample of 1,054 Australians aged 18 years and over by a social research data collection agency conducting online omnibus surveys every fortnight (I-view, North Sydney, Australia). The question included in the survey was: “Which one of the following methods do you typically use to cook beef sausages?” and response options covered grilling, pan frying, barbecuing, baking, boiling and casseroling. Pan frying and barbecuing were the two most popular methods, reported by 68% of respondents, and were selected for investigation.

For the composite cooked samples, similar cooking conditions were used as outlined above, although cooking time and temperature varied depending on the size and characteristics of the sausages. Between one and seven whole sausages (minimum mass before cooking 148 g) from each purchase were cooked without added fat or oil, either by pan-frying or barbecuing. For the lower fat sausages, longer pan-frying times at a lower heat setting were required to achieve an acceptable end product. Across all sausages, internal temperatures in cooked sausages were higher in barbecued sausages (mean 90 °C) than in pan-fried sausages (79 °C).

### 2.6. Analytical Methods

Methods of analysis were as described in previous studies of the nutrient profile of Australian meats [8,12], with the exception of methods for carbohydrates, dietary fibre, ash, iodine, alpha tocopherol, cholecalciferol and tryptophan. Table 1 outlines the methods of analysis used for these nutrients. All analyses were conducted at the National Measurement Institute (Melbourne, Australia), an ISO 9001 certified laboratory, with the exception of analysis of starch, dietary fibre, vitamin B12, folates, pantothenates and tryptophan, which were undertaken by sub-contracted laboratories. All methods, other than for tryptophan, were undertaken in laboratories accredited to conduct that analysis by the National Association of Testing Authorities (NATA).

**Table 1:** Methods of analysis for analyses not described in Williams *et al.* (2007) [8] or Droulez *et al.* (2006) [12].

Nutrient	Method Description	Reference Method	Limit of Reporting
**Sugars (fructose, glucose, sucrose, maltose, lactose)**	Aqueous extract of sample is filtered and analysed by HPLC using amino column with acetonitrile/water mobile phase containing salt, with refractive index detection	NMI in-house method VL295, based on AOAC 18th edition [13] method 31.138-31.142	0.2 g/100 g
**Starch**	Enzymatic hydrolysis to glucose, detection and quantification by spectrophotometry	DTS in-house method S6 v.1, based on AOAC 18th edition [13], 996.11 and other publications	0.1 g/100 g
**Dietary fibre**	Enzymatic-gravimetric measurement of total dietary fibre	DTS in-house method F5 v.1, based on AOAC 16th edition [14], Method 985.29	0.5 g/100 g
**Ash**	Burning of all organic material at 525 °C; gravimetric measurement of remaining inorganic matter	NMI in-house method VL286 v.5.1 based on AOAC 16th edition [14], method 923.03 and 900.02	0.1 g/100 g
**Alpha tocopherol**	Saponification, purification using solid phase extraction, then measurement using normal phase HPLC on a silica column, with detection by fluorescence with excitation at 292 nm and fluorescence at 326 nm	NMI in-house method VL291_Ver. 7.1 based on AOAC 17th edition [15] method 992.03 and other publications	0.1 mg/100 g
**Cholecalciferol**	Saponification, purification using solid phase extraction, then measurement using a normal phase LC and any vitamin D &/or 25-hydroxyvitamin D identified by Ion Trap MS-MS	NMI in-house method VL392_Ver. 1.3 based on Jakobsen *et al.* [16] and other publications	0.1 µg/100 g
**Folates, total**	Triple enzyme microbiological assay with chicken pancreas for conversion of poly-glutamates to di-glutamates, and incubation with *Lactobacillus casei*	RPH in-house method based on Davis *et al.* [17]	3 µg/100 g
**Iodine**	Extraction with tetramethyl ammonium hydroxide, then ICPMS	NMI in-house method VL345 v4.0, based on Fecher *et al.* [18]	0.1 µg/100 g
**Tryptophan**	Alkaline hydrolysis, neutralisation, derivatisation, then separation and quantification using ultra performance liquid chromatography with UV detection at 260 nm	APAF in-house method QAAA-02	2 mg/100 g
**Fatty acids**	Capillary Gas Chromatography with “SUPELCO” SP-2560 non-bonded biscyanopropyl polysiloxane column (100 m × 250 µm × 0.2 µm), hydrogen carrier gas, flame ionisation detection; identification of individual acids based on retention time against known fatty acid methyl esters including both cis and trans isomers	Extraction and derivitization as per Droulez *et al.* [12]	0.1% of methyl esters

APAF = Australian Proteome Analysis Facility; DTS = Dairy Technical Services (Melbourne); NMI = National Measurement Institute (Melbourne); RPH = Royal Perth Hospital (Perth); all laboratories based in Australia.

### 2.7. Statistical Analysis

In order to understand factors that may influence the nutrient composition of retail beef sausages, results for moisture, protein and total fat in raw sausages were compared with a two-sample independent *t*-test, using Statistical Analysis System version 9.2 (SAS, Cary, NC, USA) or Microsoft Excel 2013. Statistical significance level was set at *p* = 0.05.

### 2.8. Presentation of Data

Nutrient values are presented in accordance with the data expression format used in NUTTAB 2010 [6]. For those analytes where results were reported as being below the limit of reporting (<LOR), this value is shown in the results tables. However for estimation of calculated and mean values, if at least one sample contained a quantifiable level of the analyte, the mean value was estimated by assigning a half LOR value to the remaining values.

Calculated values, including total sugars, energy, retinol equivalents and niacin equivalents and mass of fatty acids, are calculated as in NUTTAB 2010. Fatty acids are reported as being monounsaturated or polyunsaturated only if all double bonds were present in the *cis* configuration. *Trans* fatty acids were reported by the laboratory only as total monounsaturated *trans* fatty acids and total polyunsaturated *trans* fatty acids.

## 3. Results

### 3.1. Composition of Raw Sausages—Individual Samples

A wide variety of sausages were available for purchase, varying in size, addition of flavouring ingredients (such as vegetable powders, beer or herbs), quality descriptors (e.g., terms such as “gourmet”, “select” or “finest”) and meat content. Only one type was described in a way that suggested its likely nutrient composition (“extra lean”). Table 2 presents the average mass and moisture, protein and fat content of samples grouped according to factors consumers may use to assist purchase decisions: labelled meat content, quality descriptor (quality *vs*. standard), and addition of flavouring ingredient. 

**Table 2:** Moisture, fat and protein contents (mean (standard deviation)) of raw beef sausages according to purchase location, labelled meat content and label description.

Category	Number Samples	Mass per Sausage (g)	Moisture Content (g/100 g)	Fat Content (g/100 g)	Protein ^1^ Content (g/100 g)
Meat content, label %					
≤75%	25	86.5 (30.4)	62.8 (3.9)	14.5 (4.0)	13.8 (1.5)
>75%	16	85.6 (22.3)	62.2 (4.1)	15.6 (3.9)	14.4 (1.5)
*p* value ^2^		0.91	0.65	0.36	0.20
Quality descriptors					
Standard	33	88.9 (29.1)	61.7 (3.8)	15.7 (3.8)	13.8 (1.4)
“Quality”	8	74.8 (13.4)	65.9 (2.6)	11.7 (3.2)	15.1 (1.8)
*p* value ^2^		0.05	0.003	0.004	0.015
Flavour descriptors					
Plain	21	80.5 (24.0)	63.0 (4.1)	14.6 (4.1)	14.0 (1.5)
Flavoured	20	92 (29.8)	62.3 (3.7)	15.2 (4.0)	14.1 (1.7)
*p* value ^2^		0.18	0.57	0.66	0.88
Overall (range)	41	86.2 (27.2) (27–174)	62.5 (3.9) (55.8–70.2)	14.9 (4.0) (7.3–22.6)	14.0 (1.5) (11.6–17.9)

^1^ Expressed as nitrogen content × 6.25. ^2^ Two-independent sample *t*-test.

Sausage mass averaged 86.2 ± 27.2 g ranging from 27 g for chipolata and cevap style sausages to 174 g per sausage and was lower in sausages labelled with descriptors indicating they were “higher quality” or extra lean. More than one type of beef sausage was available for purchase in each outlet visited.

Total fat was the most variable of the three nutrients measured in raw sausages. Fat content averaged 14.9 ± 4.0 g/100 g but ranged from 7.3 to 22.6 g/100 g. Sausages labelled with descriptors indicating they were “quality” (*n* = 8) had a 25% lower fat content than other sausages (*p* = 0.004). The addition of flavouring ingredients or variation in meat content, as reported on product labels or by butchers, did not influence fat content.

Protein content (average 14.0 ± 1.5 g/100 g) and moisture content (62.5 ± 3.9 g/100 g) were less variable than fat content and were also influenced by quality but not by meat content or presence of flavouring ingredients. “Quality” sausages had approximately 10% more protein (per 100 g) than standard sausages. They also had higher moisture contents, suggesting that their lower fat content is achieved by the use of leaner meat, higher in protein and moisture.

### 3.2. The Effect of Cooking Method on Moisture, Protein and Fat Content

Table 3 shows the effect of boiling, pan-frying and barbecuing on sausage mass and on moisture, protein and fat contents, in one type of sausage. While there were small differences in nutrient profile depending on cooking method, these differences were most pronounced when comparing moisture content. Boiling produced little change in the moisture content of sausages compared to raw sausages. In contrast, moisture content decreased with dry heat cooking (pan-frying and barbecuing) and there was a consequent small increase in fat and protein contents per 100 g sample. Boiling led to an increase in fat content and decrease in protein content, which possibly reflects movement of water soluble proteins and carbohydrates into the cooking water. Sausage mass changed little with boiling but decreased with the dry-heat cooking (mean 11.7% loss from raw). Overall, the two dry heat cooking methods did not have markedly different effects on sausage moisture, protein and fat contents and their most pronounced difference from raw sausages was in moisture content.

**Table 3:** Moisture, fat and protein contents of one type of beef sausage cooked by three methods.

Cooking Method	Mass per Sausage (g) (% Loss on Cooking)	Moisture Content (g/100 g)	Fat Content (g/100 g)	Protein ^1^ Content (g/100 g)
Uncooked	86.2	57.3	19.1	12.8
Boiled	88.4 (−0.6%)	58.0	22.3	11.9
Pan-fried	78.6 (10.6%)	54.9	22.1	14.0
Barbecued	76.6 (12.9%)	53.6	20.4	14.3

^1^ Expressed as nitrogen content × 6.25.

### 3.3. Nutrient Profile of Sausages Composited on Fat Content—Raw and Cooked

After consideration of the findings of analysis of raw sausages, as described above, beef sausages were assigned to one of three groups for further analysis, based on fat content, as this was the most variable nutrient and, potentially, the one most affected by formulation variation. Table 4 shows the sausage mass, proximate nutrient levels, tryptophan and cholesterol content of composite samples of beef sausages (raw, pan-fried and barbecued).

**Table 4:** Proximate composition, energy, tryptophan and cholesterol content, per 100 g edible portion, of composite samples ^1^ of beef sausages grouped according to fat content; raw, pan-fried without oil and barbecued without oil.

Cooking Method & Sausage Type	Mass per Sausage (g) (% Cook Loss)	Moisture (g)	Fat, Total (g)	Protein ^2^ (g)	Sugars, Total ^3^ (g)	Starch (g)	Dietary Fibre (g)	Ash (g)	Energy (kJ)	Tryptophan (mg)	Cholesterol (mg)
Raw sausages ^4^:											
Lower fat	74 ^5^	67.0	10.0	14.7	0.6	3.0	<0.5	2.7	683	148	60
Medium fat	80	63.9	13.8	14.2	0.5	2.2	<0.5	2.3	800	126	50
Higher fat	92	58.7	18.8	13.5	0.7	2.2	<0.5	2.2	976	98	52
Mean	84	62.5	14.9	14.0	0.6	2.4	<0.5	2.4	843	120	53
Pan-fried sausages											
Lower fat	69 (7%) ^4^	63.1	13.2	16.4	0.6	3.4	<0.5	2.6	837	134	61
Medium fat	71 (9%)	61.5	14.5	17.0	0.4	3.1	0.5	2.7	889	132	54
Higher fat	80 (11%)	56.8	18.5	16.0	0.7	3.1	0.6	2.4	1020	141	54
Mean	74 (10%)	60.1	15.8	16.5	0.6	3.2	0.5	2.6	929	136	56
Barbecued sausages											
Lower fat	64 (13.5%) ^4^	61.8	11.3	17.3	0.6	3.2	<0.5	2.7	778	162	71
Medium fat	71 (14.7%)	61.5	15.4	17.4	0.4	3.4	<0.5	2.6	932	148	58
Higher fat	75 (19%)	56.6	18.1	16.8	0.7	3.6	0.7	2.7	1030	168	56
Mean	71 (16%)	59.7	15.6	17.1	0.6	3.4	0.4	2.7	938	159	60

^1^ There were 16 samples in each of the medium- and higher-fat composites and 9 in the lower fat composite. Mean values are weighted according to these sample numbers. All sausages were 100% edible; ^2^ Expressed as nitrogen content × 6.25; ^3^ Sum of fructose, glucose and sucrose; no maltose or lactose was present in any samples; ^4^ Moisture, protein and fat are predicted content based on analysis of individual samples before freezing. Remaining values are based on analysis of composite samples; ^5^ Raw sausage mass is the average across sausages before cooking by either method. The weight loss values are calculated only from the mass of the sausages cooked by that method.

A comparison of predicted and analysed levels of moisture, protein and fat in the composite samples of raw sausages showed that the lower fat sausages lost considerable moisture during frozen storage prior to compositing (predicted: 67 g/100 g, analysed: 62.2 g/100 g) and consequently levels of fat and protein were higher than predicted (fat analysed: 12.8 g/100 g, 28% increase; protein analysed: 16.4 g/100 g, 12% increase) (see Supplementary Table S1). This effect was also seen in the medium and higher fat sausage but was less marked than in the lower fat sausages (medium fat: moisture 0.7 g/100 g lower; higher fat: moisture 0.5 g/100 g lower than predicted). In the presentation of results, the analysed moisture, fat and protein contents of composite raw sausages have been replaced with the values estimated from the composition of the individual sausages included in these composite samples. However it was not possible to predict the effects of this moisture loss on levels of other nutrients in raw sausages, nor in cooked sausages, and no adjustments have been made to these values.

As the fat content of the raw composite samples decreased, protein content increased. Levels of carbohydrates and ash were similar in all samples regardless of fat content. The main sugar present was glucose, with lower levels of sucrose and fructose in some samples. No maltose or lactose was detected in any samples. Carbohydrate and fibre levels reflect the addition of cereal-based pre-mixes to sausages. Despite having lower total fat contents, the lower fat sausages had higher cholesterol levels. Williams *et al.* [8], in a study of lean Australian raw beef, reported cholesterol levels ranging from 35 to 76 mg/100 g depending on the cut selected.

Table 5 shows the vitamin content of beef sausages. Carotenes present in the sausages would be contributed largely by the addition of spices and other coloured foods, such as tomato, although some would arise from the meat fat. A notable finding was the presence of cholecalciferol in most samples. This is consistent with recent findings for Australian beef [19]. Niacin equivalents reflect substantial levels of both pre-formed niacin and tryptophan, which can be metabolized to form niacin [20].

Table 6 shows mineral levels in beef sausages. Sodium levels are high, resulting from addition of salt and some sodium from additives such as sodium phosphates, as well as natural occurrence in meat. Sodium was higher in the lower fat sausages, suggesting additional salt is used to improve palatability. The low levels of iodine in all samples indicates that salt used is not iodised. Zinc levels were slightly higher in the lower fat sausages, which possibly reflects the use of leaner meat in these sausages, as beef lean contains more zinc than beef fat [6].

There was little variation in fatty acid composition between samples, even comparing lower fat to higher fat sausages, where lower levels of saturated fatty acids might have been expected [12] (see Table 7). None of the sausages sampled contained ingredients, other than beef, that would contribute significant amounts of fat. The fatty acid profiles found are consistent with that of Australian beef, particularly beef separable fat, although somewhat lower in polyunsaturated and *trans* fatty acids than recent published data [12]. Low levels of the omega-3 fatty acid docosapentaenoic acid were found in the higher fat raw sausages, as has regularly been reported for Australian beef [13], which is predominantly grass fed. However levels of this fatty acid were below the limit of reporting in the lower fat and medium fat raw samples. Supplementary Table S2 contains all fatty acid data reported by the laboratory.

**Table 5:** Vitamin (“Vit”) content of composite samples of beef sausages ^1^ grouped according to fat content; raw, pan-fried without oil and barbecued. All values are expressed per 100 g edible portion.

Cooking Method & Sausage Type	Vitamin A ^2^				Vit B1	Vit B2	Vitamin B3 ^2^		Vit B5	Vit B6	Vit B12	Vit D	Vit E	Folates
	Alpha Carotene (µg)	Beta Carotene (µg)	Retinol (µg)	Retinol Equiv (µg)	Thiamin (mg)	Riboflavin (mg)	Niacin (mg)	Niacin Equiv. (mg)	Panto-thenate (mg)	Pyrid-oxine (mg)	Cobalamin (µg)	Cholecalci-ferol (µg)	Alpha Tocopherol (mg)	Total Folate (µg)
Raw sausages														
Lower fat	87	230	35	81	0.03	0.09	4.1	6.6	0.26	0.11	2.8	0.6	0.3	<3
Medium fat	28	190	58	92	0.02	0.08	3.7	5.8	0.15	0.09	2.2	0.6	0.4	<3
Higher fat	43	170	23	55	0.02	0.07	3.6	5.2	0.14	0.10	1.8	0.8	0.4	<3
Mean	47	190	39	75	0.02	0.08	3.8	5.7	0.17	0.10	2.2	0.7	0.4	<3
Pan-fried sausages														
Lower fat	75	220	25	68	<0.02	0.09	3.6	5.8	0.32	0.06	2.8	0.5	0.4	<3
Medium fat	25	130	32	56	<0.02	0.08	4.2	6.4	0.28	0.06	3.0	<0.5	0.6	<3
Higher fat	52	160	21	52	<0.02	0.07	3.3	5.7	0.22	0.07	2.6	0.6	0.5	<3
Mean	47	160	26	57	<0.02	0.08	3.7	6.0	0.26	0.06	2.8	0.4	0.5	<3
Barbecued sausages														
Lower fat	150	380	36	112	<0.02	0.08	4.2	6.9	0.32	0.06	3.2	<0.5	0.4	<3
Medium fat	30	140	24	50	<0.02	0.09	4.4	6.9	0.29	0.07	3.1	0.6	0.5	10.6
Higher fat	65	170	22	56	<0.02	0.07	4.0	6.8	0.23	0.06	2.8	1.0	0.6	<3
Mean	70	200	26	66	<0.02	0.08	4.2	6.9	0.27	0.06	3.0	0.7	0.5	5.2^3^

^1^ There were 16 samples in each of the medium- and higher-fat composites and 9 in the lower fat composite. Mean values are weighted according to these sample numbers. All sausages were 100% edible; ^2^ Retinol equivalents = retinol + beta carotene/6 + alpha carotene/12. Niacin equivalents = niacin + tryptophan/60. Tryptophan values presented in Table 4. No ascorbic acid was found in any sample; ^3^ Estimated by assigning half LOR value to those sausages whose folates level was reported as <LOR.

**Table 6:** Mineral content of composite samples of beef sausages ^1^ grouped according to fat content; raw, pan-fried without oil and barbecued. All values are expressed per 100 g edible portion.

Cooking Method & Sausage Type	Calcium (mg)	Iodine (µg)	Iron (mg)	Magnesium (mg)	Phosphorus (mg)	Potassium (mg)	Selenium (µg)	Sodium (mg)	Zinc (mg)
Raw sausages									
Lower fat	10	3	1.4	18	200	280	7	840	3.2
Medium fat	19	6	1.5	15	180	220	7	680	2.8
Higher fat	9	2	1.0	14	170	220	5	700	1.8
Mean	13	4	1.3	15	180	230	6	720	2.5
Pan-fried sausages									
Lower fat	12	3	1.4	18	200	260	8	800	3.4
Medium fat	11	9	1.5	17	200	250	8	780	3.2
Higher fat	11	4	1.2	16	200	250	6	770	2.4
Mean	11	5	1.4	17	200	250	7	780	2.9
Barbecued sausages									
Lower fat	12	3	1.5	19	210	270	9	830	3.7
Medium fat	11	9	1.6	18	200	260	10	830	3.2
Higher fat	14	3	1.4	18	210	270	6	810	2.8
Mean	12	5	1.5	18	210	270	8	820	3.2

^1^ There were 16 samples in each of the medium- and higher-fat composites and 9 in the lower fat composite. Mean values are weighted according to these sample numbers. All sausages were 100% edible.

**Table 7:** Fatty acid profile ^1^ of composite samples ^2^ of raw and cooked ^3^ beef sausages grouped according to fat content. Values are expressed as g/100 g total fatty acids ^4^.

Fatty Acid (% of Total Fatty Acids)	Lower Fat		Medium Fat		Higher Fat		Mean	
	Raw	Cooked	Raw	Cooked	Raw	Cooked	Raw	Cooked
C10:0	0.1	0.1	<0.1	<0.1	<0.1	0.1	<0.1	0.1
C12:0	0.1	<0.1	<0.1	0.1	<0.1	0.1	<0.1	0.1
C14:0	2.8	2.8	3.1	3.1	3.8	3.6	3.3	3.2
C15:0	0.6	0.6	0.5	0.6	0.6	0.6	0.6	0.6
C16:0	25.1	25.4	26.0	25.8	26.8	26.1	26.1	25.8
C17:0	1.6	1.5	1.5	1.5	1.5	1.4	1.5	1.5
C18:0	18.5	18.0	18.4	18.1	17.7	17.4	18.1	17.8
C20:0	0.2	0.2	0.2	0.2	0.1	0.1	0.2	0.2
C24:0	0.1	0.1	0.2	0.1	<0.1	<0.1	0.1	0.1
Total saturates (%)	49.1	48.7	49.9	49.5	50.5	49.4	49.9	49.4
C14:1	0.6	0.6	0.7	0.8	0.9	0.8	0.8	0.8
C16:1	3.5	3.5	3.8	3.8	4.0	4.0	3.8	3.8
C18:1	39.5	39.9	38.5	38.9	37.8	38.6	38.4	39.0
C20:1	0.2	0.3	0.2	0.2	0.2	0.2	0.2	0.2
Total monounsaturates (%)	43.8	44.2	43.2	43.8	42.9	43.6	43.2	43.8
C18:2n6	1.5	1.7	1.5	1.5	1.5	1.6	1.5	1.6
C18:3n3	0.5	0.5	0.4	0.5	0.5	0.5	0.5	0.5
C20:3n6	0.1	0.1	0.1	0.1	<0.1	0.1	0.1	0.1
C20:4n6	0.2	0.2	0.2	0.2	<0.1	<0.1	0.1	0.1
C22:5n3	<0.1	0.2	<0.1	0.2	0.1	0.1	<0.1	0.2
Total polyunsaturates (%)	2.3	2.7	2.2	2.5	2.1	2.3	2.2	2.5
Total monounsaturated trans	2.5	2.5	2.6	2.5	2.5	2.7	2.5	2.6
Total polyunsaturated trans	0.3	0.2	0.5	0.2	0.2	0.2	0.3	0.2
Total trans fatty acids (%)	2.8	2.7	3.1	2.7	2.7	2.9	2.8	2.8

^1^ The following fatty acids were not quantifiable in any samples: C4:0, C6:0, C22:0, C17:1, C18:3n6, C20:2n6, C20:3n3, C20:5n3, C22:2n6, C22:4n6, C22:6n3; ^2^ There were 16 samples in each of the medium- and higher-fat composites and 9 in the lower fat composite. Mean values are weighted according to these sample numbers. All sausages were 100% edible; ^3^ Cooked values are the average of values for panfried and grilled sausages for the respective group; individual values are in Table S2; ^4^ Fatty acid totals may not add to 100% as they include only those fatty acids able to be quantified.

### 3.4. Effect of Cooking on Nutrient Profile of Composite Samples

All sausages lost mass when pan fried or barbecued, as expected, but the loss was less in the lower fat sausages than in the two higher fat groups. Loss was greater in the barbecued sausages than in the pan-fried ones, for each fat category, which reflects the higher internal temperature achieved in these sausages. Weight loss on cooking in the composite samples was similar to that found in the study of a single type of sausage and resulted from loss of both moisture and, in most cases, fat. As a result, there was a concentration in protein and carbohydrate contents. Cholesterol levels did not drop during cooking, in spite of the loss of some fat.

Levels of niacin, panthothenate and cobalamin increased during cooking, on a wet weight basis, suggesting there was no substantial destruction of these vitamins during cooking, with the increase in concentration a result of water and fat loss. Levels of other vitamins remained constant, with the exception of retinol, pyridoxine and thiamin, where there were substantial declines in concentration as a result of cooking.

For minerals, no major changes as a result of cooking were noted beyond a concentration effect through loss of moisture and fat. Fatty acid profile also did not appear to be influenced substantially by cooking (see Supplementary Table S2).

The overall nutrient profiles of pan-fried and barbecued sausages were very similar and there were no notable differences between these two cooking methods.

### 3.5. Comparison to Existing NUTTAB Nutrient Data for Beef Sausages

Table 8 compares selected data found in this study for raw sausages to the corresponding record held in NUTTAB 2010 [6]. The key difference between the new data and the older NUTTAB records is that, on average, sausages have become lower in fat and higher in protein since the NUTTAB data were generated. The NUTTAB data aligns more closely with the higher fat composite sample, suggesting that in the period since the NUTTAB data were generated, a more diverse product range has become available that covers leaner as well as more “traditional” formulations.

The higher levels of carotenes in the samples in this survey are likely to reflect the now-common use of ingredients such as dried vegetables and spice extracts to add flavor to sausages. Some differences in vitamin levels between the new and older data were found and may reflect changes in analytical methods over this time rather than other major formulation changes. Iron levels are lower than reported for the oldest NUTTAB data but similar to that of the more recent NUTTAB grilled sausage data (1.3 mg/100 g). The iron levels found in this survey are consistent with a meat content of around 75%, based on the most recent data on iron in lean Australian beef [8]. Although sodium levels in raw sausages appear higher than in the NUTTAB records, for the cooked sausages the levels are similar and also consistent with recent data from a national survey of 94 samples of beef sausages (780 mg/100 g) [5]. No major differences in fatty acid profiles (% of total fatty acids) were noted between these samples and the NUTTAB data.

**Table 8:** Comparison of selected nutrients, in raw sausages from this survey and from NUTTAB, and in a serve of raw lower fat beef sausages compared with raw, beef rump steak (separable lean).

Nutrient	Units	Sausages, Raw, Per 100 g				Per Serve, Raw ^3^		
		Lower Fat	Medium Fat	Higher Fat	NUTTAB ^1^	Lower Fat Sausages	Rump Steak, Separable Lean	Amount for Source (Good Source) Claim ^4^
Energy	kJ/100 g	683	800	976	1060	1010	855	-
Protein	g/100 g	14.7	14.2	13.5	12.5	21.8	38.8	5 (10)
Total fat	g/100 g	10.0	13.8	18.8	21.4	14.8	5.3	-
Total saturates	g/100 g	4.7	6.5	8.9	9.9	6.9	1.9	-
Carotenes ^2^	µg/100 g	317	218	213	54	470	19	-
Niacin, preformed	mg/100 g	4.1	3.7	3.6	2.6	6.1	5.7	1 (2.5)
Vitamin A ^2^	µg /100 g	81	92	55	26	120	4	75 (188)
Vitamin B12	µg /100 g	2.8	2.2	1.8	-	4.1	2.5	0.2 (0.5)
Iron	mg/100 g	1.4	1.5	1.0	1.6	2.1	4.0	1.2 (3.0)
Phosphorus	mg/100 g	200	180	170	160	296	437	100 (250)
Selenium	µg /100 g	7	7	5	-	10	15	7 (18)
Sodium	mg/100 g	840	680	700	650	1240	95	-
Zinc	mg/100 g	3.2	2.8	1.8	2.5	4.7	7.8	1.2 (3.0)

^1^ NUTTAB records 08E20116 Sausage, beef, raw; 08A10761Beef, rump steak, separable lean, raw. “Separable lean” refers to the meat flesh with all visible fat removed; ^2^ Vitamin A expressed as retinol equivalents, including alpha and beta carotene and retinol; ^3^ Serve regarded as two raw sausages (148 g) or a 190 g portion of raw steak, equivalent to two serves of meat [21]; ^4^ See [22] and [23].

## 4. Discussion

This survey showed that there is a wide range of beef sausages available for purchase in Australia with substantial differences in fat content, with the highest fat raw sample (22.6 g/100 g) containing three times the fat content of the lowest fat sample (7.3 g/100 g). There was less variability in protein content between sausages available for purchase.

As a result of this greater diversity of beef sausages, the overall mean fat content of raw sausages in this survey is 30% lower than in samples analysed in 1988, and consequently saturated fat levels are also reduced and protein contents are, on average, 10% higher. Whilst traditional style, higher fat sausages are similar in fat content to sausages available for purchase in 1988, more recent “lean” and “quality” beef sausages are significantly lower in fat, possibly due to use of leaner meat. The main nutrient-related effect of dry heat cooking was to reduce moisture content, as has been found in other studies [24]. There was little difference between pan-frying and barbecuing on nutrient levels and future surveys of sausage composition could reduce costs by investigating only one of these popular cooking methods.

The levels of vitamins and minerals found in this study are generally similar to those found in earlier studies as reported in NUTTAB 2010 [6], taking into account likely changes in analytical techniques over this time. However a notable difference was the higher level of carotenes found in this study, possibly resulting from addition of flavouring ingredients such as vegetable powders. Of particular note is that this study is the first to have reported levels of cholecalciferol in Australian sausages, using a technique suitable for measuring lower levels in unfortified foods [19]. It is also likely that sausages would contain measurable levels of 25-hydroxy cholecalciferol, which has vitamin D activity and has recently been reported in Australian beef [19].

Sodium levels remain high in beef sausages and do not appear to have dropped since 1988 despite considerable action to reduce sodium levels in Australian foods [25]. A previous survey of sodium levels in barbecued beef sausages, in 2011, found that they ranged widely, from 322 to 1660 mg/100 g, averaging 780 mg/100 g [5]. This suggests that there is some potential to reduce sodium levels in beef sausages while still maintaining palatability and microbial safety.

Australian nutrition labelling standards require certain levels of vitamins, minerals and protein to be present in a serve of a food before claims that the food is a source (at least 10% of the Recommended Dietary Intake as set out in Standard 1.1.1 [23], or 5 g protein [22]) or good source (at least 25% RDI or 10 g protein) of that vitamin or mineral. Raw lower fat sausages, in this survey, averaged 74 grams, and it is reasonable to assume that an adult would consume two of these in a meal. At this serve size, sausages in this study were found to be good sources of protein, vitamin B12, niacin, zinc and phosphorus, and a source of vitamin A (as retinol equivalents), iron and selenium.

Although beef sausages remain, on average, higher in fat than lean cuts of beef, the finding that some sausages had much lower fat contents indicates it is possible to make lower fat sausages that are acceptable to consumers. Manufacturers who do this may be able to make appropriate nutrition labelling claims for these products. The current requirement for foods to be labelled as reduced in fat is that the reduction in fat is at least 25% [22] and this is already being met by both the lower fat group of raw sausages (46% less fat than the higher fat raw sausages) and the medium fat group (26% lower than higher fat sausages). Using the average fat content found in this survey (14.9 g/100 g) as the comparator, there were already sausages sampled where fat content was 25% below this level.

Comparing lower fat raw sausages to raw rump steak (muscle only) (see Table 8), a serve of two sausages delivers more vitamin A, niacin and vitamin B12, and lower but still important levels of protein, iron, phosphorus, selenium and zinc. The significant levels of key nutrients found in sausages in this survey suggest they have the potential to form part of a varied main meal repertoire when served with vegetables, potatoes or wholegrain choices. According to the Australian Guide to Healthy Eating [21], fresh sausages may count as a serve as part of the “Lean meats and poultry, fish, eggs, tofu, nuts and seeds, and legumes/bean” group, if they are salt and fat reduced and made mostly from lean meat.

A strength of this study was the large number of samples of sausages included, covering the diverse range of styles now available in Australia and the major outlets where they are purchased. The sample size was much larger than was used to generate NUTTAB data (between 5 and 10 purchases) [6]. However in a future survey it would be beneficial to collect samples from a larger number of retail outlets and over a broader time period to ensure that the diverse range of sausage making styles is fully represented. A challenge in this survey, and in any future surveys, was the need to purchase and transport samples to the laboratory in good condition and in time to prepare samples and to store them so that spoilage did not occur. The loss of moisture from the lower fat sausages after freezing indicates the difficulties that can arise from necessary sample handling processes, which can influence the results obtained.

The findings of this study suggest that further research on sausages made from other types of meat, such as pork, chicken and lamb, would be useful to determine if existing data for their nutrient composition remains current.

## 5. Conclusions

Australian beef sausages vary widely in composition but are, on average, leaner and higher in protein than reflected in existing data in the national food composition tables, NUTTAB, although sodium levels have not changed substantially. This survey shows that sausages have the potential to provide important levels of key nutrients and that lower fat formulations can be successfully made. The new nutrient values generated in this survey will be used to update NUTTAB and to support product labelling and consumer education.

## Supplementary Materials

Supplementary materials can be accessed at: http://www.mdpi.com/2072-6643/7/11/5491/s1. Table S1. Comparison of estimated *vs*. analysed values for mean moisture, fat and protein levels in composite samples of raw sausages. Table S2. Fatty acid profiles of composite samples of beef sausages.
